# Healing capacity of bone marrow mesenchymal stem cells versus platelet-rich fibrin in tibial bone defects of albino rats: an *in vivo* study

**DOI:** 10.12688/f1000research.15985.1

**Published:** 2018-09-28

**Authors:** Dina Rady, Rabab Mubarak, Rehab A. Abdel Moneim

**Affiliations:** 1 Department of Oral Biology, Faculty of Dentistry, Cairo University, Cairo, 11553, Egypt

**Keywords:** bone regeneration, bone marrow derived mesenchymal stem cells, platelet rich fibrin.

## Abstract

**Background: **Various techniques for tissue engineering have been introduced to aid the regeneration of defective or lost bone tissue. The aim of this study was to compare the *in vivo* bone-forming potential of bone marrow mesenchymal stem cells (BM-MSCs) and platelet-rich fibrin (PRF) on induced bone defects in rats’ tibiae.

**Methods: **In total, one defect of 3-mm diameter was created in each tibia of 36 Wistar male rats. There were two groups: group A, left tibia bone defects that received PRF; and group B, right tibia bone defects of the same animal that received BM-MSCs loaded on a chitosan scaffold. Subsequently, Scanning electron microscope/energy-dispersive X-ray (SEM/EDX) analyses was performed at 3 and 10 days, and 3 weeks post‑implantation and following euthanasia; (n=12).

**Results: **The EDX analysis performed for each group and time point revealed a significant increase in the mean calcium and phosphorous weight percentage in the BM-MSC-treated group relative to the PRF-treated group at all-time intervals (P < 0.05). Moreover, the mean calcium and phosphorus weight percentage increased as time progressed since the surgical intervention in the PRF-treated and BM-MSCs groups (P < 0.05).

**Conclusions: **In the present study, both BM-MSCs and PRF were capable of healing osseous defects induced in a rat tibial model. Yet, BM-MSCs promoted more adequate healing, with higher mean calcium and phosphorous weight percentages than PRF at all-time points, and showed greater integration into the surrounding tissues than PRF.

## Introduction

Several biomaterials are used to treat bone deficiencies ^1^. Autologous bone graft limitations are related to harvesting process including the quality and quantity of grafted bone and complications at the second surgical site, while allogenic bone grafts carry the risk of disease transmission and immunological rejection. Hence, there are considerable motivations for developing alternative solutions for bone regeneration ^2^. The use of tissue engineering approaches has proven to be effective in inducing bone formation by applying mesenchymal stem cells (MSCs) ^3^ or platelet-rich fibrin (PRF) ^4^. The capacity of bone marrow mesenchymal stem cells (BM-MSCs) for bone repair has been well reported *in vivo* with promising results; BM-MSCs remain the most widely used source of osteogenic cells in bone tissue engineering studies ^5– 7^ MSCs are undifferentiated cells capable of replication ^8^ that have the potential to differentiate along multiple cell lineages, giving rise to cells that form mesenchymal tissues, including bone, cartilage and muscle ^9^. PRF is a second-generation platelet-rich biomaterial ^10^. PRF is derived from a natural and slowly progressive polymerization process occurring during centrifugation, which increases incorporation of the circulating cytokines and growth factors in the fibrin mesh and prevents them from undergoing proteolysis ^11^. In addition, the PRF fibrin matrix provides an optimal support for MSCs which constitute the determining elements responsible for real therapeutic potential ^12, 13^. Platelets are active growth factor-secreting cells that initiate wound-healing, connective tissue healing and cell proliferation ^14^. Therefore, PRF is considered as an inexpensive autologous fibrin scaffold prepared in approximately one minute and hence no cost for membrane and bone graft ^15^. In the present research, rats were used as they are easy to handle and less expensive. In addition, breeding cycles are substantially shorter, providing enough animals in a reasonable amount of time ^16^. Research on bone tissue engineering is focused on the development of alternatives to autologous bone grafts for bone reconstruction. Although multiple stem cell-based products and biomaterials are currently being examined, comparative studies are rarely achieved to evaluate the most appropriate approach in this context. The purpose of this study was to compare the regenerative capacity of bone marrow (BM)-MSCs and PRF implanted in surgically induced bone defects in rats’ tibiae.

## Methods

### Ethical statement

The study protocol was approved by the Research ethics committee of Faculty of Dentistry, Cairo University (151031).

### Experimental procedure

A total of 36 male Wistar rats weighing 175–200 g, aged 12–14 weeks-old were used in this study. The animals were obtained from and housed in the Animal house, Faculty of Medicine, Cairo University. The animals were randomly placed in separate cages under controlled room temperature 25±2°C with 12/12 h light/dark cycle and were fed food and water *ad libitum*.

### Isolation, culture and identification of BM-MSCs

BM-MSCs were isolated from the femurs of 6 Wistar donor (6-weeks-old male) rats (100±20 g), BM-MSCs isolation and propagation occur in 14 days before experimental procedures under aseptic conditions as previously described ^17^. Briefly, bone marrow was harvested by flushing the femurs with Dulbecco’s modified Eagle’s medium (DMEM, GIBCO/BRL) supplemented with 10% fetal bovine medium (GIBCO/BRL). Cells were isolated with a density gradient [Ficoll/Paque (Pharmacia)] and cultured in culture medium supplemented with 1% penicillin-streptomycin (GIBCO/BRL) at 37°C in a humidified 5% CO _2_ incubator. When large colonies developed (80–90% confluence), cultures were washed twice with phosphate buffer saline (PBS) and cells were trypsinized with 0.25% trypsin in 1 mM EDTA (GIBCO/BRL) for 5 minutes at 37°C. After centrifugation (at 2400 rpm for 20 minutes), cells were re-suspended with serum-supplemented medium and incubated in 50 cm ^2^ culture flask Falcon. On day 14, the adherent colonies of cells were trypsinized, and counted. ^17^. Cultures confluence was monitored by inverted light microscope (Olympus, USA) with a digital camera (Nikon, Japan).

Surface antigens CD90 and CD34 were detected by flow cytometry to allow identification of BM-MSCs as follows. Following blocking in 0.5% BSA and 2% FBS in PBS, 100,000 cells were incubated in the dark at 4°C for 20 min with the following monoclonal antibodies; FITC CD 90 (PN IM1839U; Beckman Coulter), PE CD 34 (PN IM1871U; Beckman Coulter, USA). Mouse isotype PE antibody (Beckman Coulter, USA) were used as controls (dilution of all antibodies, 1:1500). Cells were washed and suspended in 500 µl fluorescence activated cell sorting (FACS) buffer and analyzed using a Cytomics FC 500 flow cytometer (Beckman Coulter, USA) using CPX software version 2.2. BM-MSCs osteogenic differentiation was induced by StemPro osteogenic induction medium; incubation for 7 days at the third passage, 1 × 10 ^3^ cells/each well by osteocyte StemPro osteogenesis differentiation kit (Gibco, Life Technology) at concentration 300 µl of osteogenic medium (stempro medium) and identified by alizarin red staining (Sigma-Aldrich) for 30 min at room temprature, the mineralized nodules were stained and monitored using inverted light microscope (Olympus, USA) with a digital camera (Nikon, Japan). The results were presented by descriptive analysis.

### Establishment of bone defects

The surgical approach was under general anaesthesia via intramuscular injection of 50-75 mg/kg ketamine chlorohydrate (Amoun CO) and 20 mg/ Kg body weight xylazine HCL (Xyla-Ject®, Phoenix ^TM^, Pharmaceutical Inc.) in the proximal–medial area of each tibia. While blood samples were being prepared, a 3-mm diameter bone defect was created using a round surgical bur ^3^ under constant irrigation with saline solution in both tibiae of the same animal (split-body design) to avoid selection bias and neutralize any confounders that may affect the outcomes of both treatments. Experimental groups were standardized among all the animals: group A, left tibia defect received PRF clot immediately placed by sterile tweezers in the defect; Group B, right tibia of the same animal received BM-MSCs seeded on chitosan scaffold then implanted in the tibial bone defect using sterile spatula. Both groups were randomly sub-divided according to time of euthanasia into three sub-groups (1, 2 and 3); at 3, 10 days and 3 weeks, respectively (n = 12) ( Table 1). Postoperatively, periosteum flaps and skin were sutured. Anti-inflammatories and antibiotics were applied on the skin and injected for 3 days. Each animal received IM 10 mg/kg flumox (Eipico, Egypt) to avoid secondary bacterial infection, 10 mg/kg cataflam (Novartis, Egypt) to relieve postoperative pain and topical antibiotic spray; Bivatracin (Egyptian Company For Advanced Pharma, Egypt) to avoid local infection. The animals were euthanized by intra-cardiac overdose of sodium thiopental (80 mg/kg).

**Table 1.** Experimental groups and sub-groups.

Groups	Sub group	Time of euthanasia
Group A	Sub-group A1	3 days postoperatively
	Sub-group A2	10 days postoperatively
	Sub-group A3	3 weeks postoperatively
Group B	Sub-group B1	3 days postoperatively
	Sub-group B2	10 days postoperatively
	Sub-group B3	3 weeks postoperatively

### Scaffold fabrication

To obtain a porous chitosan scaffold to deliver BM-MSCs into the defect, 1 g chitosan (Merck Germany) was dissolved in 200 µl 0.2% M acetic acid , stored for 1 day at room temperature, poured into a 3-mm diameter stainless steel circular mould, stored in deep freezer at −70°C for 5 days, then lyophilized for 3 days as follows. In the lyophiliser (Thermo Fisher Scientific), there were three phases of preparation. The first phase was freezing phase, where the sample was exposed to −40°C in a vacuum for 10 min. The second phase was warm up vacuum pump phase, where sample was exposed to −15°C in a vacuum for 20 min. The third phase was main drying phase, where sample was exposed to 30°C in a vacuum for 3 days; after the 3 days, a blank porous chitosan scaffold was prepared ^18, 19^.

### Preparation of BM-MSCs

Prior to cell seeding, the lyophilized scaffolds were immersed in absolute ethanol for sterilization. Hydration was accomplished by sequential immersion in serially diluted ethanol solutions of 70, 50, and 25%, during intervals of 30 min each. Scaffolds were finally equilibrated in PBS followed by standard culture medium (30 min; 3 times), and then placed in tissue culture plates ready to be seeded. BM-MSCs were seeded at a density of 2.5x10 ^6^ cells/scaffold under static conditions, by means of a cell suspension. The seeded scaffold was then placed in the defect to deliver the stem cells to the defect, which was then sutured closed.

### Preparation of PRF

A total of 2 ml venous blood was drawn from the caudal vein of rats used in the experiment into a plain tube and immediately centrifuged at room temperature with a lab centrifuge (Electronic centrifuge 800, China) for 10 min at 3000 rpm ^20^. In the middle of the tube, a fibrin clot was formed between the supernatant acellular plasma and the lower red corpuscles. The PRF clot was detached using sterile tweezers applied to the bone defect.

### Scanning electron microscopy/energy-dispersive X-ray (SEM/EDX) analysis

Tibiae were carefully dissected free from soft tissue; bone specimens of each group were sectioned using a disc in low speed hand piece under constant irrigation to include the entire defect sites. Specimens were placed in 2.5% buffered glutaraldehyde solution (pH 4.7) for 6 hours. Then, dehydrated in increasing concentrations of ethanol (50, 70, 85, 95 and 100%) for 10 minutes at each concentration. Finally, they were mounted on EM stubs and examined using SEM (Model Quanta 250 FEG FEI Thermo Fisher Scientific, USA, with accelerating voltage 30 K.V., magnification14–1,000,000× and resolution for Gun.1n) *.* The EDX analysis system works as an integrated feature of SEM Quanta FEG 250 attached with EDX unit, (FEI Company, Netherlands). EDX analysis of the bone surfaces was performed, the elemental distribution of phosphorus and calcium (expressed as weight percentage) were determined. Composition scans were collected at randomly selected points in the bone surfaces of the defect using the backscattered electron mode. Data were obtained by calculating the mean of ten independent determinations ^21^.

### Statistical analysis

One-way analysis of variance (ANOVA) was used to compare different observation times within the same group. This was followed by Tukey’s post hoc test when the difference was found to be significant. A t-test was used to compare between both groups using IBM SPSS 18.0 version 21 for windows (SPSS Inc., Chicago, IL, USA). The significance level was set at p ≤ 0.05.

## Results

### *In vitro* evaluation of BM-MSCs

**Figure 1.**
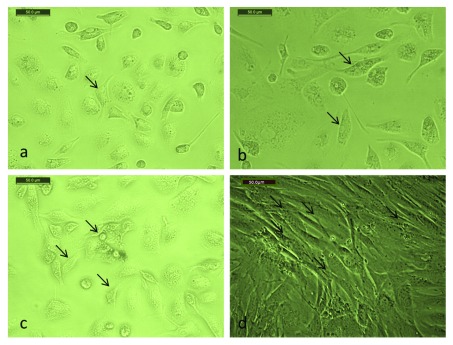
BM-MSCs in culture at different durations. ( **a**) Cells cultured for 3 days (x100). ( **b**) Cells cultured for 7 days (x100). ( **c**) Cells cultured for 10 days (x100). ( **d**) Cells cultured for 14 days (x100).

**Figure 2.**
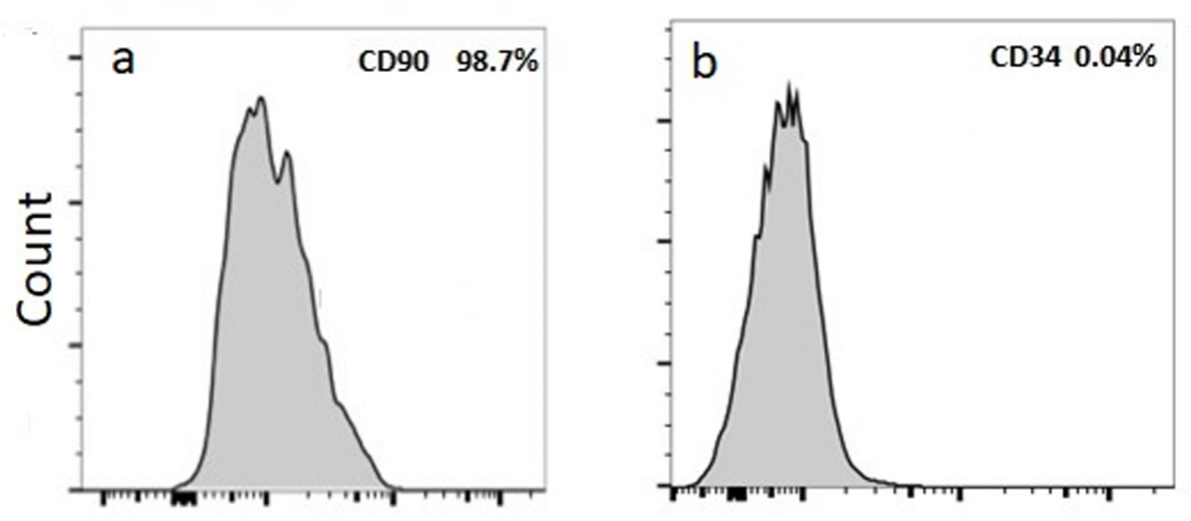
FACS analysis of bone marrow mesenchymal stem cells. ( **a**) Positive for CD90 (98.7%). ( **b**) Negative for CD34 (0.04%).

**Figure 3.**
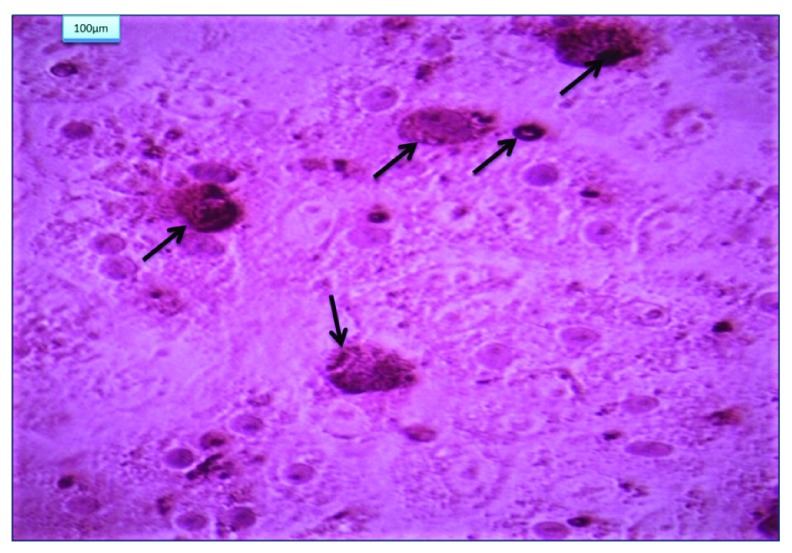
Bone marrow mesenchymal stem cells (arrows) differentiated into osteoblasts and stained with Alzarin red (x200).

Cells were rounded non-adhesive isolated bone marrow cells ( Figure 1a). Adhesive cells started to get fusiform spindle shaped fibroblast like cells. Cell confluence was 30–40% ( Figure 1b). Cells became adhesive fusiform spindle shaped fibroblast like cells. Cell confluence was 50–70% ( Figure 1c). Cells were long and spindle-shaped, reaching the highest confluence (90%) at 14 days ( Figure 1d). BM-MSCs were negative for CD34 and positive for CD90 (>98.7%) ( Figure 2). BM-MSCs were positively stained with alizarin red ( Figure 3).

### SEM/EDX analysis

**Figure 4.**
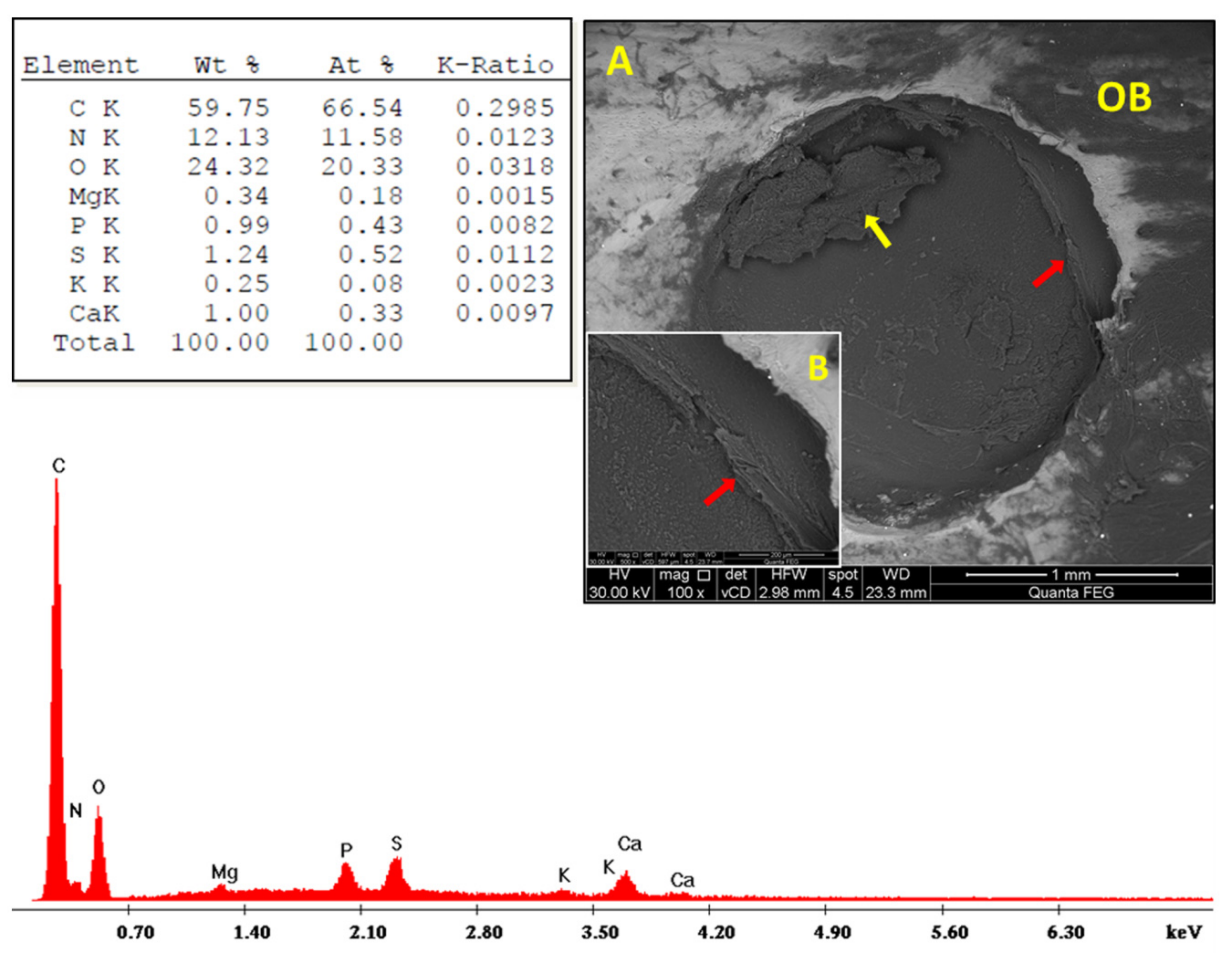
Scanning electron microscopy/energy-dispersive X-ray analysis sub-group A1 showing: PRF traces (red arrows), fibro-cellular tissue (yellow arrow) and old bone (OB). ( **A**) x100; ( **B**) x500.

**Figure 5.**
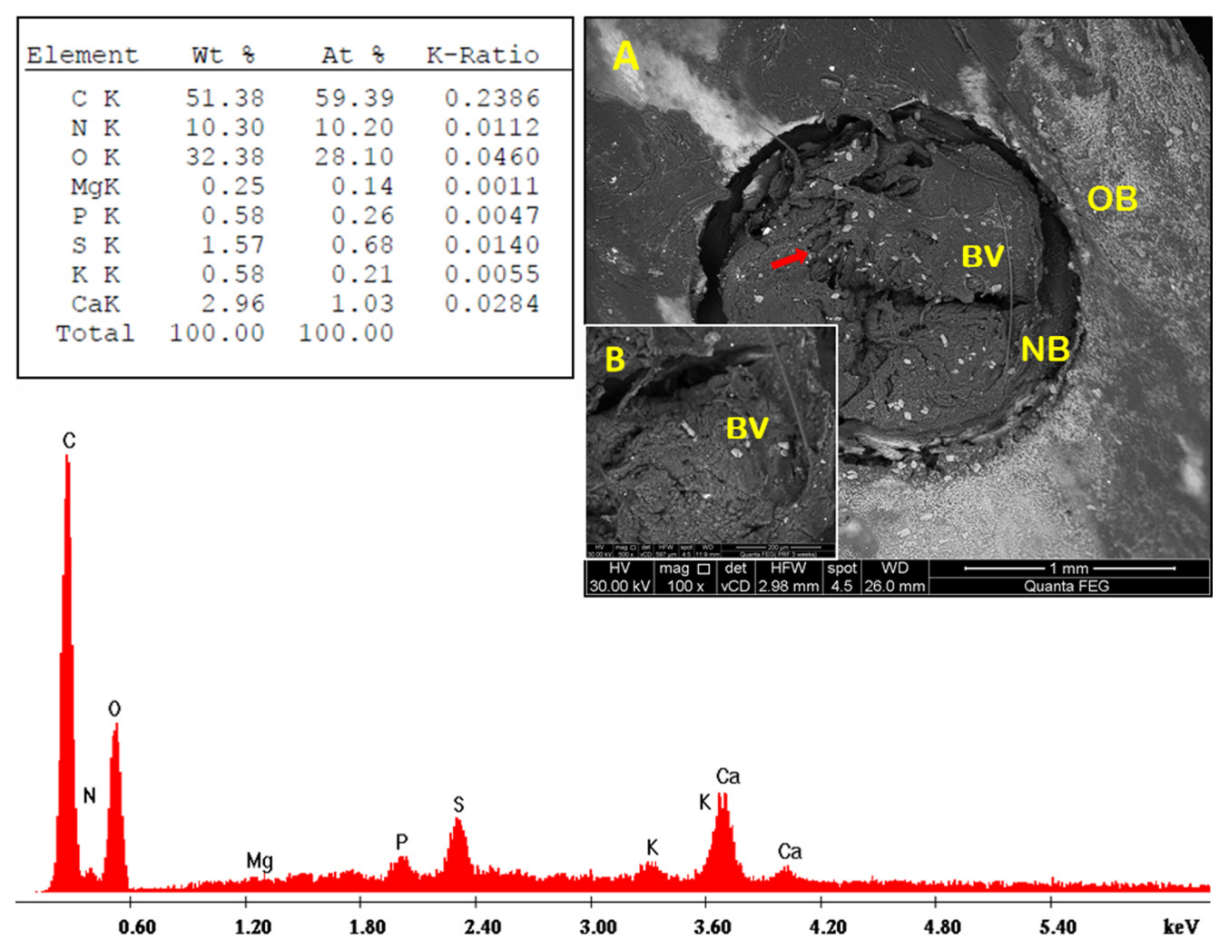
Scanning electron microscopy/energy-dispersive X-ray analysis sub-group A2 showing: new bone (NB), blood vessels (BV), PRF traces (red arrow) and old bone (OB). ( **A**) x100; ( **B**) x500.

**Figure 6.**
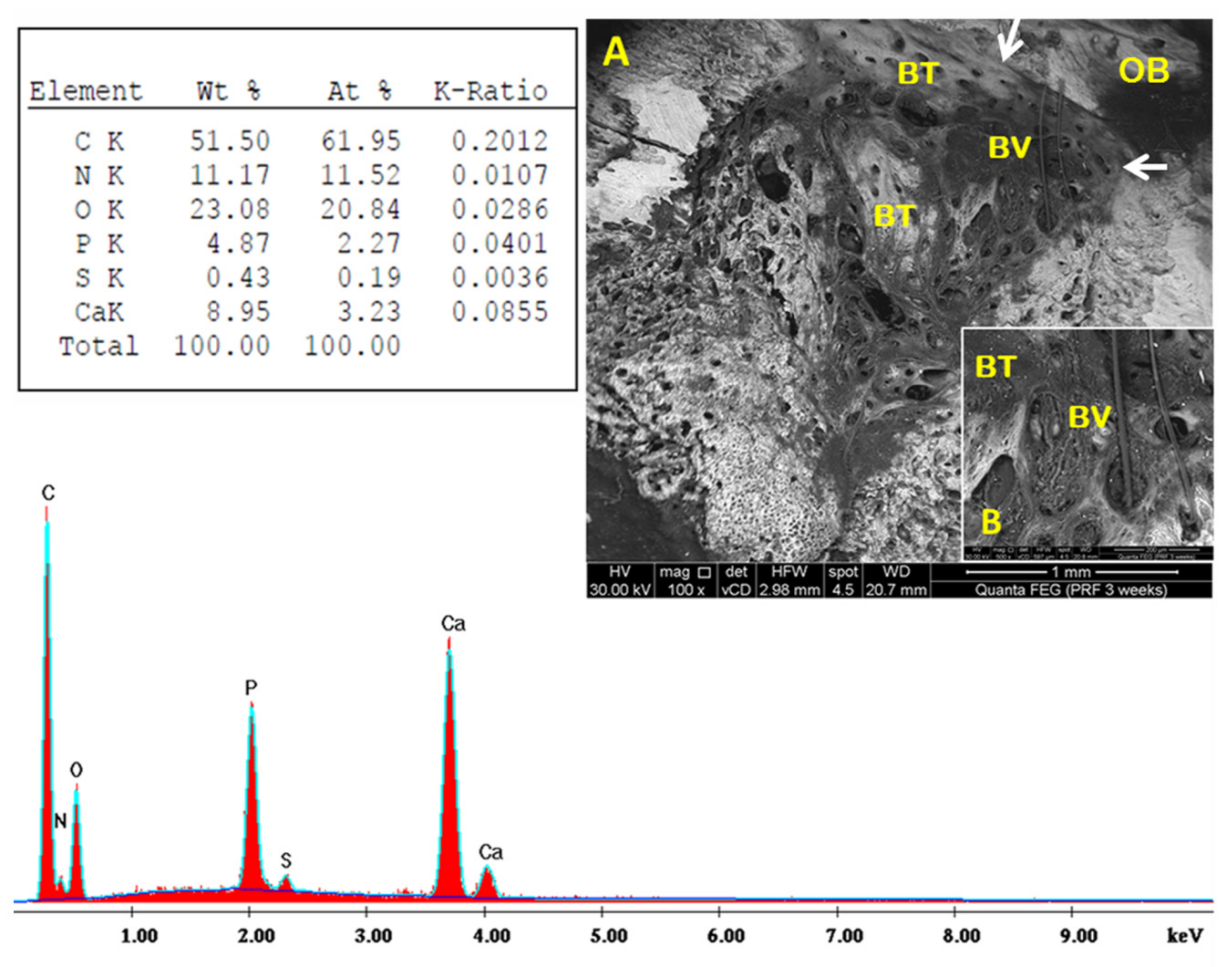
Scanning electron microscopy/energy-dispersive X-ray analysis sub-group A3 showing bone trabeculae (BT), and blood vessels (BV). Note the borders between the newly formed bone and the cortical surroundings were no longer detectable (white arrow). ( **A**) x100; ( **B**) x500.

**Figure 7.**
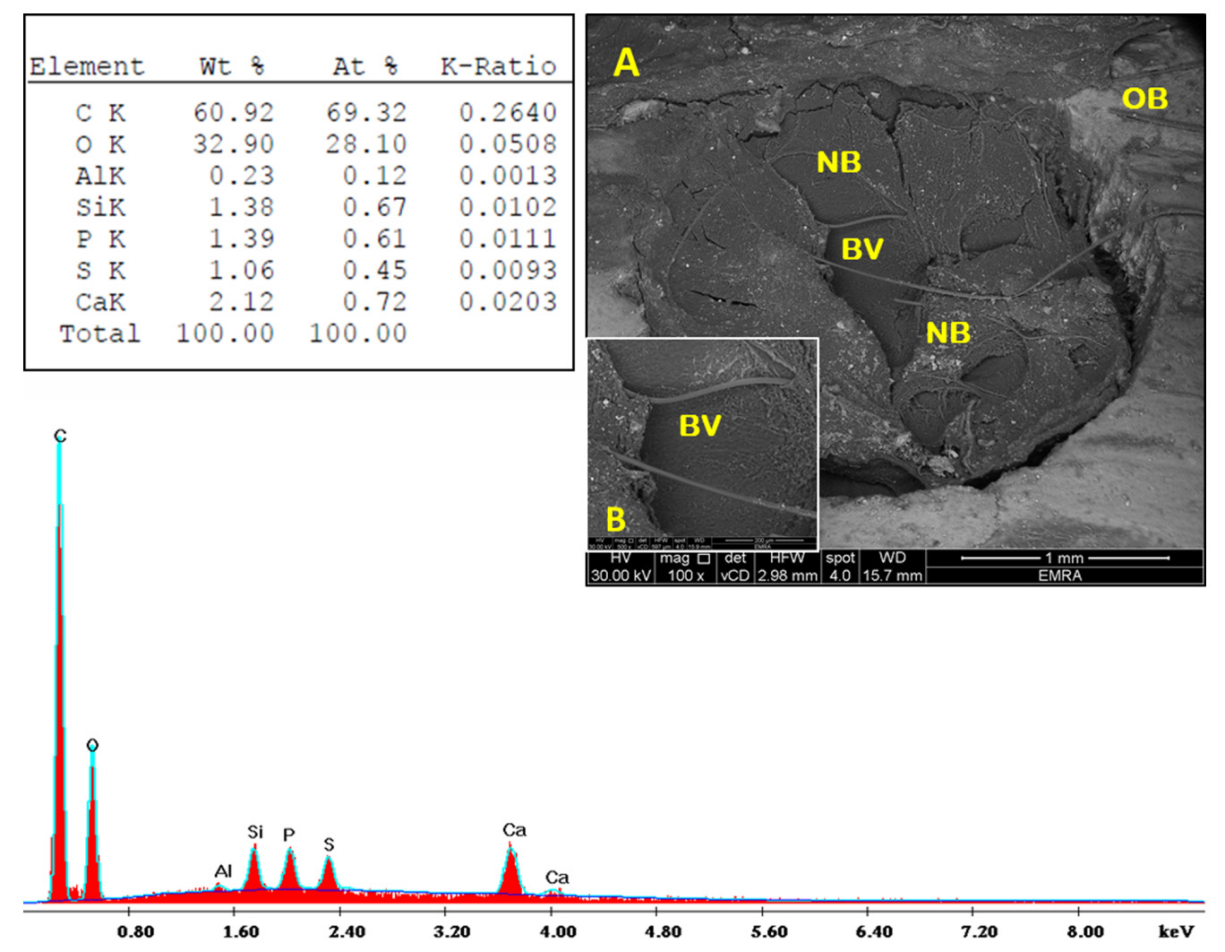
Scanning electron microscopy/energy-dispersive X-ray analysis sub-group B1 showing new bone (NB), blood vessels (BV) and old bone (OB). ( **A**) x100; ( **B**) x500.

**Figure 8.**
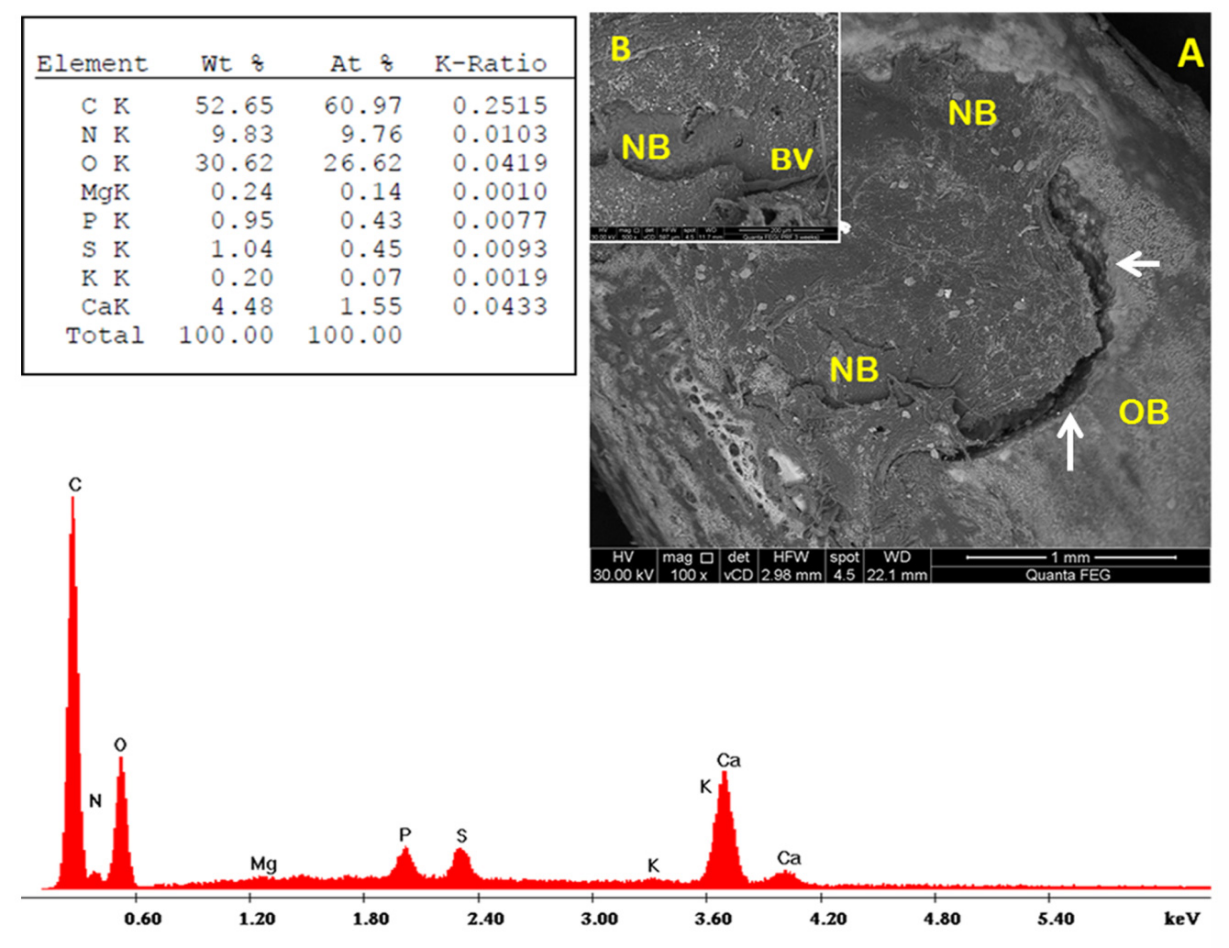
Scanning electron microscopy/energy-dispersive X-ray analysis sub-group B2 showing new bone (NB), blood vessel (BV) and old bone (OB). Note the interface between new and old bone (white arrows). ( **A**) x100; ( **B**) x500.

**Figure 9.**
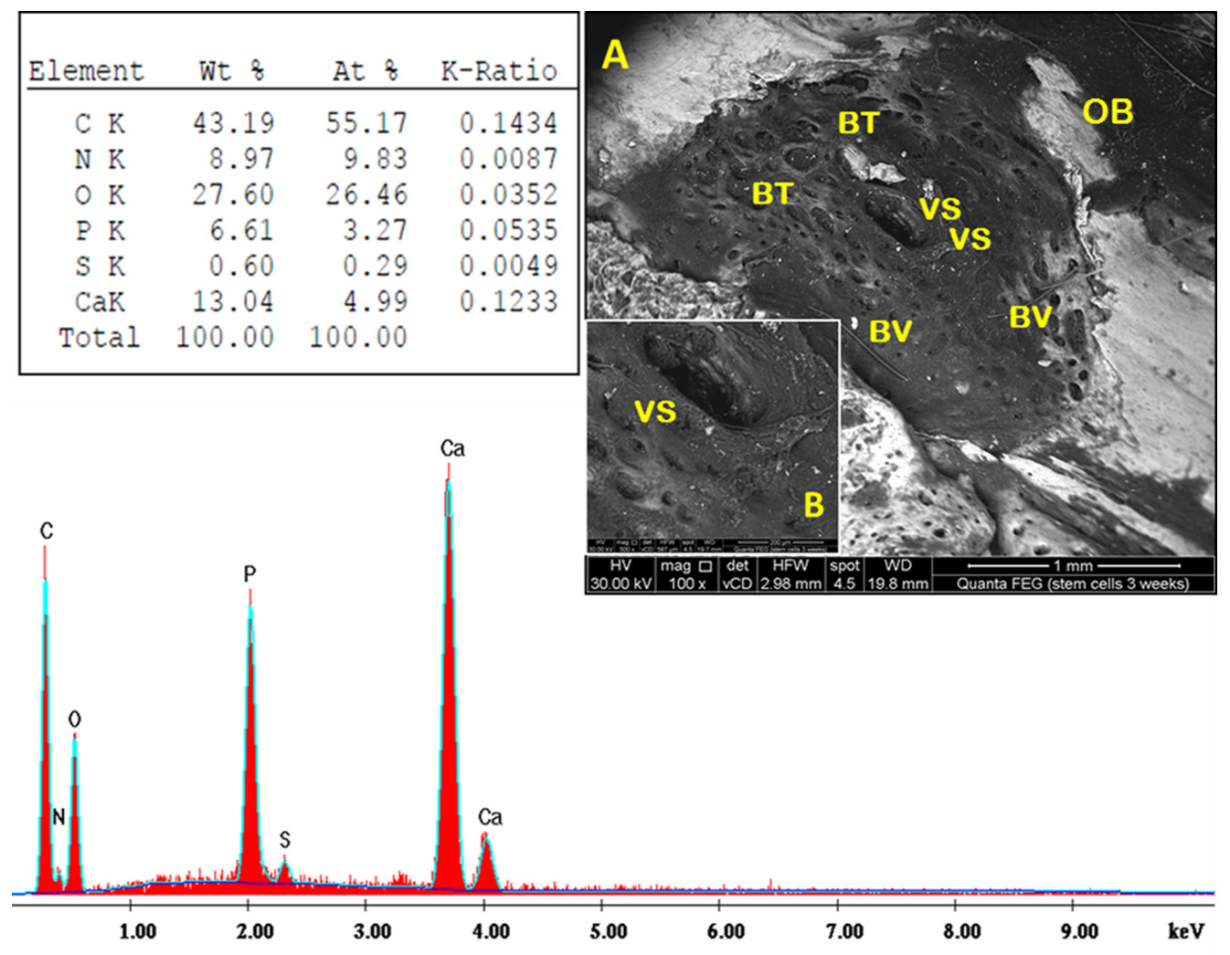
Scanning electron microscopy/energy-dispersive X-ray analysis sub-group B3 showing bone trabeculae (BT), blood vessels (BV), vessel-space (VS) and old bone (OB). Note the more integrated interface between new and old bone. ( **A**) x100; ( **B**) x500.

Fibro-cellular tissue and traces of PRF material were seen in sub-group A1 ( Figure 4). In sub-group A2, along the margins, bone was actively forming; blood vessels were seen to be emerging and inserted close to the newly formed bone. PRF remnants were observed in the defect centre ( Figure 5). Sub-group A3 revealed spongy-like pattern with abundant non-remodelled vascular spaces containing fibro-cellular tissue. In addition, bone formation extended beyond the perimeter of the original defect site compared to sub-group A2 ( Figure 6). In sub-group B1, numerous vessels appeared, along with dis-organized architecture of newly formed bone ( Figure 7). The bone in sub-group B2 was nearly restored, with new bone extending partly beyond the perimeter of the defect ( Figure 8). Sub-group B3 revealed more organized bone architecture; well-oriented thick and smooth interconnecting bone trabeculae with large-centred vascular space. Many blood vessels were noticed. The borders between the newly formed bone and the pre-existing old bone were almost remodeled; no longer detectable in most areas and cannot be distinguished from the cortical surroundings ( Figure 9). Raw EDX data are shown in Dataset 1 ^22^.

### Statistical results

**Figure 10.**
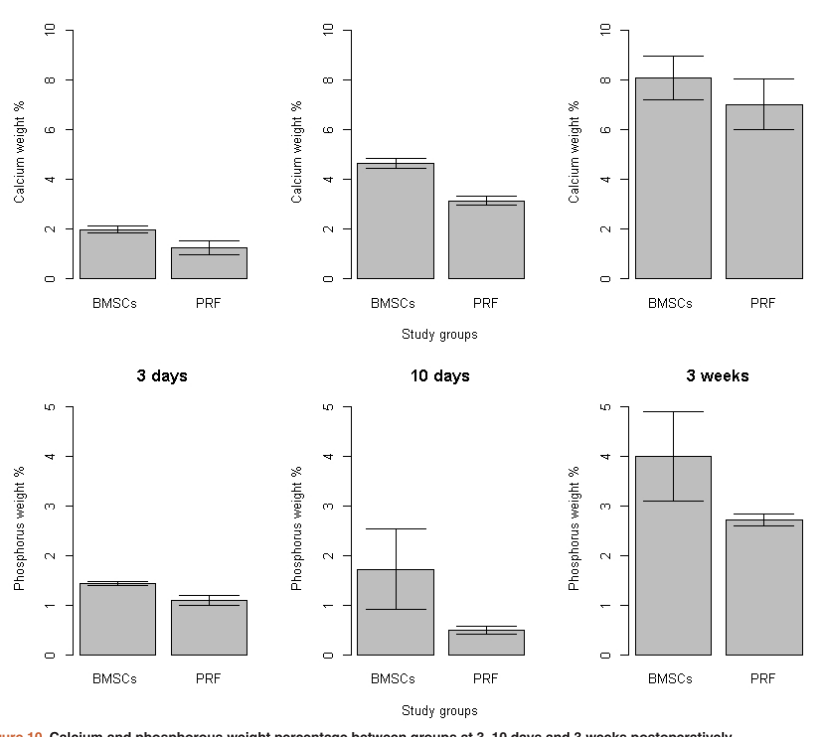
Calcium and phosphorous weight percentage between groups at 3, 10 days and 3 weeks postoperatively.

There was significant increase in the mean calcium and phosphorous weight percentage of group B relative to group A at all-time intervals. Moreover, calcium and phosphorus weight percent mean value increased by time in both groups ( Figure 10 and Table 2– Table 5).

**Table 2.** Calcium weight percentage between groups.

Postoperative time of euthanasia	Groups	Mean	SD	95% CI		t	P
				Lower	Upper		
3 days	Group A	0.74	0.40	0.49	0.99	6.46	<0.0001
	Group B						
10 days	Group A	1.52	0.01	1.51	1.52	1004.94	<0.0001
	Group B						
3 weeks	Group A	1.08	0.16	0.98	1.18	23.88	<0.0001
	Group B						

**Table 3.** Calcium weight percentage for each group at different observation times.

Groups	Postoperative time of euthanasia	Mean	SD	95% CI		Min	Max	F	P
				Lower	Upper				
Group A	3 days	1.25 ^c^	0.26	1.08	1.42	1.00	1.50	266.07	<0.0001
	10 days	3.15 ^b^	0.19	3.02	3.27	2.96	3.33		
	3 weeks	7.03 ^a^	1.03	6.37	7.69	6.04	8.02		
Group B	3 days	1.99 ^c^	0.14	1.90	2.08	1.86	2.12	411.54	<0.0001
	10 days	4.66 ^b^	0.19	4.54	4.78	4.48	4.84		
	3 weeks	8.11 ^a^	0.88	7.55	8.67	7.27	8.95		

Tukey’s post hoc test: mean values with different superscript letters are significantly different from each other.

**Table 4.** Phosphorous weight percentage between groups.

Postoperative time of euthanasia	Group	Mean	SD	95% CI		t	P
				Lower	Upper		
3 days	Group A	0.93	0.04	0.90	0.96	77.11	<0.0001
	Group B						
10 days	Group A	0.63	0.92	0.05	1.21	2.37	<0.0001
	Group B						
3 weeks	Group A	1.29	0.79	0.78	1.79	5.65	<0.0001
	Group B						

**Table 5.** Phosphorous weight percentage for both groups in different observation times.

Groups	Postoperative time of euthanasia	Mean	SD	95% CI		Min	Max	F	P
				Lower	Upper				
Group A	3 days	0.50 ^c^	0.08	0.45	0.55	0.42	0.58	1475.9	<0.0001
	10 days	1.10 ^b^	0.11	1.03	1.16	0.99	1.20		
	3 weeks	2.72 ^a^	0.11	2.65	2.79	2.61	2.83		
Group B	3 days	1.43 ^b^	0.04	1.40	1.46	1.39	1.47	48.531	<0.0001
	10 days	1.73 ^b^	0.81	1.21	2.24	0.95	2.50		
	3 weeks	4.01 ^a^	0.90	3.43	4.58	3.14	4.87		

Mean values with different superscript letters are significantly different from each other.

Raw data for EDX analysis and flow cytometry gating graphs for identification of BM-MSCs Also included are raw SEM images Click here for additional data file. Copyright: © 2018 Rady D et al. 2018 Data associated with the article are available under the terms of the Creative Commons Zero "No rights reserved" data waiver (CC0 1.0 Public domain dedication).

## Discussion

Bone regeneration using BMSCs was very well reported and standardized in many protocols, Donzelli *et al.*, 2007 showed that the adult rat bone marrow was a suitable source for MSCs that can be easily induced to differentiate into an osteogenic lineage, so they are thought to be a promising candidate, supporting cells for bone reconstruction ^23^. Most in *vitro* and many *in vivo* studies have proposed that MSCs possess the ability to increase osteoinduction and osteogenesis ^24– 27^.

Through the current study, BM-MSCs and PRF promoted bone regeneration; where the newly formed bone was almost remodelled and integrated into the surrounding old bone with well-vascularized fibro-cellular tissue. In addition, evidence of osteogenesis was reflected by the presence of blood vessels. However, there was an improved bone regenerative capacity in defects treated with BM-MSCs compared to those treated with PRF. SEM-EDX analysis revealed a significant increase in the mean calcium and phosphorous weight percentage in the BM-MSCs group relative to the PRF group at all-time intervals.

Considering PRF, growth factors released are postulated to be promoters of tissue regeneration, tissue vascularity, mitosis of MSCs and osteoblasts and rate of collagen formation, playing a key role in the rate and extent of bone formation ^28^. This would help explain the significant increase in calcium and phosphorus weight percentage in the PRF group through the experiment.

Accordingly, there was a marked drop in elemental analysis of calcium and phosphorous in sub-group A1, that increased gradually in sub-groups A2 and A3. This can be explained by the findings of a previous study on the pattern of growth factor release where PRF sustained long-term release of TGF-1 and PDGF-AB that peaked at day 14, which led to increase mineralization then decreased mildly but had a delayed peak of release ^11^.

The BM-MSC-treated group exhibited more organized bone architecture than the PRF-treated group; sub-group B3 exhibited well-oriented thick and smooth interconnecting bone trabeculae filling the defect compared to sub-group A3; which revealed spongy-like pattern with abundant non-remodelled vascular spaces containing fibro-cellular tissue. The proposed mechanism through which BM-MSCs contribute to bone regeneration enhancement is via maturation into osteoblasts *in vivo*, or via an indirect pathway by paracrine effects on host stem or progenitor cells ^29^. Notably, a significant increase in the mean calcium and phosphorus weight percentage was observed in BM-MSC-treated group at all time intervals throughout the experiment when compared with the corresponding PRF group. In accordance with the findings of a previous study, SEM/EDX analysis of osteogenic differentiated MSCs seeded on collagen scaffold demonstrated that calcium co-localized with phosphorous, with a gradual increase of both chemical elements observed from day 7 up to very high levels at day 28 ^23^. In conclusion, we confirmed that PRF yielded inferior bone formation to that by BM-MSCs after implantation in rat tibiae.

## Data availability

The data referenced by this article are under copyright with the following copyright statement: Copyright: © 2018 Rady D et al.

Data associated with the article are available under the terms of the Creative Commons Zero "No rights reserved" data waiver (CC0 1.0 Public domain dedication). 

Dataset 1. Raw data for EDX analysis and flow cytometry gating graphs for identification of BM-MSCs. Also included are raw SEM images. DOI: https://doi.org/10.5256/f1000research.15985.d218548 ^22^.
