# Supplementary material for: Healing capacity of bone marrow mesenchymal stem cells versus platelet-rich fibrin in tibial bone defects of albino rats: an in vivo study
Source: F1000Res. 2018 Sep 28;7:1573. [Version 1] doi: 10.12688/f1000research.15985.1 (PMC6173118; doi:10.12688/f1000research.15985.1)
Supplement: Raw data for EDX analysis and flow cytometry gating graphs for identification of BM-MSCs — Also included are raw SEM images [file f1000research-7-17459-s0000.tgz › 339f9f8c-4d48-4901-8228-6f203c93a82f_Data.zip!/Data/SEM raw.doc]

**Sub- group A1 (100x)**

**Sub- group A1 (500x)**

**Sub-group A2 (100X)**

**Sub-group A2 (500x)**

**Sub-group A3 (100x)**

**Sub-group A3 (500x)**

**Sub-group B1 (100x)**

**Sub-group B1 (500x)**

**Sub-group B2 (100x)**

**Sub-group B2 (500x)**

**Sub-group B3 (100x)**

**Sub-group B3 (500x)**
