# Supplementary material for: Healing capacity of bone marrow mesenchymal stem cells versus platelet-rich fibrin in tibial bone defects of albino rats: an in vivo study
Source: F1000Res. 2018 Sep 28;7:1573. [Version 1] doi: 10.12688/f1000research.15985.1 (PMC6173118; doi:10.12688/f1000research.15985.1)
Supplement: Peer review history

# Peer review: Referee response for version 1

**DOI:** 10.5256/f1000research.17459.r38850

**Published:** 2018-10-04

**Authors:** Mahmoud M. Bakr

**Affiliations:** 1 School of Dentistry and Oral Health, Griffith University, Nathan, Qld, Australia

Overall the study was well performed and showed some interesting results. After looking at referees' reports which summarized some revisions required for this paper, I would like to add the following:

1. A statement needs to be added in the methods outlining the power analysis performed to determine the sample size in each group.
2. Despite the localized effect of BM-MSCs and PRF being applied on a scaffold, the authors should add in their discussion the potential systemic effect of both treatments especially that the choice was made to have both defects created in the same animal (Right and left tibis with different treatments). I am guessing that this was done to reduce the number of animals used in the experiment. However, the systemic effect of treatments should be addressed as a limitation of the study and/or a discussion should made to clarify how the systemic effect could not have contributed to the significant results.
3. Statistical analysis: A two ANOVA analysis should be more approriate to use in order to investigate the single main effects of time and treatment as well as the possible interaction between treatment and time. This will add more strength to the results rather than using post hoc results and t-tests.

I have read this submission. I believe that I have an appropriate level of expertise to confirm that it is of an acceptable scientific standard.

# Peer review: Referee response for version 1

**DOI:** 10.5256/f1000research.17459.r38851

**Published:** 2018-10-02

**Authors:** Reham Magdy Amin

**Affiliations:** 1 Oral Biology Department, Faculty Of Dentistry, Ain Shams University, Cairo, Egypt

The Idea of research article  

 "Healing capacity of bone marrow mesenchymal stem cells versus platelet-rich fibrin in tibial bone defects of albino rats: an *in vivo* study" by Rady et al., is interesting and valuable as it is comparing  two different ways for enhancing bone defect healing in vivo  which could be a base for clinical application .

 Overall, the paper is clear, substantially easy to read and well constructed but still, there are suggested minor comments the authors could deal with, or at least discuss for additional impact. 

**Methods**

- It is worthy mentioning the number of rats per cage 
- Further details  for clarifying the methodology and aseptic conditions of  BM-MSCs isolation from the femurs of donor rats  will be a valuable add.
- It is recommended to mention how the depth of  bone defect was controlled to be standard  in all experiment induced defects .
- It is an add to mention the place of the lab where the  BM-MSCs isolation procedures were done. 
- You need to give further details about  the size and methodology of BM-MScs and  PRF pellet  loading  in the bone defect 

**Results**

- Fig. 3 need show monitoring of  the positively  stained calcified nodules  as mentioned in the methodology
- Recommendations would be worth mentioning  depending on the paper results 

I have read this submission. I believe that I have an appropriate level of expertise to confirm that it is of an acceptable scientific standard.

# Peer review: Referee response for version 1

**DOI:** 10.5256/f1000research.17459.r38852

**Published:** 2018-10-01

**Authors:** Mohamed Shamel

**Affiliations:** 1 Oral Biology Department, Faculty of Dentistry, Modern Sciences and Arts University, Cairo, Egypt

First of all, I would like to congratulate the authors for attempting to undertake this project which I found very interesting and of valuable additional knowledge. The manuscript itself is well-written and well-structured.

 The authors of this study have investigated the Healing capacity of bone marrow mesenchymal stem cells versus platelet-rich fibrin in tibial bone defects of albino rats. Based on the study results, the authors have concluded that  BM-MSCs promoted more adequate healing, with higher mean calcium and phosphorous weight percentages than PRF at all-time points, and showed greater integration into the surrounding tissues than PRF.

 However, the authors need to address the following minor remarks:

**Methods:**

 The auhtors mentioned "experimental procedures under aseptic conditions as previously described", however I found no previously described information.

**Results:**

 1. In vitro evaluation of BM-MSCs: It is not clear in the text the shape of cells which were found belongs to which group, 3 days or 7 days?

 2. Alizarin red staining was used however no sufficient information was provided for the benefits of using this stain.

**Discussion**:

 Discussion of the results is quite comprehensive. In analyzing the results, the authors also show citations from the previous study to support the explanation of these results.

**Conclusions:**

 I think the authors should have added more points to conclude the hard work they have done.

I have read this submission. I believe that I have an appropriate level of expertise to confirm that it is of an acceptable scientific standard.

# Peer review: Referee response for version 1

**DOI:** 10.5256/f1000research.17459.r38853

**Published:** 2018-10-01

**Authors:** Mahmoud Al Ankily

**Affiliations:** 1 Oral Biology Department, Faculty of Dentistry, British University in Egypt, Cairo, Egypt

This report by Rady et al., examines the healing capacity of bone marrow mesenchymal stem cells versus platelet-rich fibrin in tibial bone defects of albino rats. the authors' inclusion of PRF yielded inferior bone formation to that by BM-MSCs after implantation in rat tibiae. The study, although it may be small, adds knowledge to the existing literature. Suggested minor comments would help to improve the impact of this paper:

 Methods

1. Isolation, culture and identification of BM-MSCs:(aseptic conditions as previously described) No previously described information found.
2. Establishment of bone defects: what is the depth of the defect? Did it reach the bone marrow spaces?
3. Preparation of PRF: taking 2ml of blood of rats used in the experiment may lead to death of the animal or affecting its healing capacity. it was more safe to use a donor like preparation of BM-MSCs.
4. It was better to compare your results with a control group to evaluate the normal healing capacity with other groups.

Results

1.  SEM/EDX analysis: Figures (B) x500 is not so clear please do not minimize them to save the magnification benefits.

I have read this submission. I believe that I have an appropriate level of expertise to confirm that it is of an acceptable scientific standard.
